# Identification, Characterization and Epitopes Prediction of an Almond Allergen Pru du 8 Fragment

**DOI:** 10.3390/ijms27062683

**Published:** 2026-03-15

**Authors:** Lihua Zhou, Kuan Gao, Changbao Hu, Weichao Zhu, Zhihui Liu, Zhihua Wu, Hongbing Chen

**Affiliations:** 1 State Key Laboratory of Food Science and Resources, Nanchang University, Nanchang 330047, China; 15679421009@163.com (L.Z.); 367900240001@email.ncu.edu.cn (K.G.); 18189830072@163.com (C.H.); 417900220189@email.ncu.edu.cn (W.Z.); 417900240123@email.ncu.edu.cn (Z.L.); chenhongbing@ncu.edu.cn (H.C.); 2 College of Food Science and Technology, Nanchang University, Nanchang 330031, China; 3 Sino-German Joint Research Institute, Nanchang University, Nanchang 330047, China; 4 Jiangxi Province Food Engineering Research Center of Special Medical Purposes Intended for Allergic Population, Nanchang 330047, China

**Keywords:** Pru du 8, purification, antibacterial protein, epitope

## Abstract

Pru du 8 is a 31 kDa antibacterial protein and officially recognized almond allergen, but its native form in mature almonds has not been characterized, and no effective extraction method has been established. In this study, we successfully isolated a high-purity (>95%) 15 kDa protein from almond extract using a one-step chromatographic approach. Mass spectrometry identified this protein as a fragment of Pru du 8. The purified 15 kDa fragment exhibited strong IgE-binding activity with sera from almond-allergic patients, and its IgE-binding specificity was confirmed by negative reactivity with non-atopic control blood serum. Structural characterization by circular dichroism and ultraviolet spectroscopy revealed that the fragment adopts a predominantly α-helical, compact fold. Furthermore, computational epitope prediction identified key B-cell epitopes on this naturally occurring fragment. This study provides the isolation method for a native Pru du 8 fragment and confirms its allergenic potential, offering a valuable tool for future research on almond allergy.

## 1. Introduction

Food allergy is defined as an adverse effect caused by a specific immune response that can recur after exposure to a particular food [1]. Tree nuts are essential components of a healthy diet; however, they are among the eight major food allergen groups recognized by international food safety and public health organizations [2]. Almonds are one of the oldest and most important nuts grown in the world [3]. At the same time, they are also tree nuts that may cause allergies [4]. Symptoms of almond allergy tend to be persistent once triggered. Mild cases may present with skin itching and rashes, and severe cases may lead to respiratory symptoms, such as asthma and breathing difficulties [5].

To date, multiple allergenic proteins have been identified in almonds. Ten principal allergenic components have been characterized: some with formal WHO/IUIS designations (Pru du 1, Pru du 3 (a non-specific lipid transfer protein 1, nsLTP1), Pru du 4 (a profilin), Pru du 5 (60S acidic ribosomal protein 2), Pru du 6 (an 11S legumin known as prunin), Pru du 8 (an antimicrobial protein with cC3C repeats), Pru du 10) and some reported in the literature but not yet formally named in the WHO/IUIS database (thaumatin-like protein (referred to as Pru du 2), almond vicilin, and almond γ-conglutin), thermal processing, such as roasting, has been reported to affect the allergenic potential of almond proteins by altering protein conformation and epitope accessibility [6]. One of these components is Pru du 8, which is formally registered in the authoritative WHO/IUIS Allergen Nomenclature Database; it is a cysteine-rich antibacterial protein with the characteristic C[X]3C−[X]10−12−C[X]3C motif of hairpin [7]. This motif can be found in the N-terminal or signaling peptides of some vicilins [8]. Notably, the allergen was first investigated over two decades ago, but sequence alignment with other food allergens belonging to the 2S albumin family had been misclassified as a 2S albumin [9]. In 2015, a computer simulation investigation based on bioinformatics analysis mistakenly identified it as almond 7S vicilin [10]. In 2019, Che et al. isolated its cDNA, the complete sequence of almond allergen Pru du 8 was obtained, and its immunoglobulin E (IgE) binding capacity was confirmed [7]. Natural Pru du 8 has not been detected or isolated from almonds [8]. Recent studies have suggest that it belongs to a distinct protein family, and its function requires further study [8]. Therefore, the isolation and purification of natural Pru du 8 is of great importance.

Antimicrobial peptides in Australian nut kernels are produced through 7S globulin processing [11], and cysteine-rich antimicrobial peptides in Impatiens seeds are derived from precursor proteins. A wheat protein homologous to an almond protein is an antimicrobial protein, and its cDNA sequence encodes a precursor protein; however, it undergoes post-translational cleavage [12]. Hence, this process might be necessary for seeds during germination process or may be regulated by plants in response to an environment subjected to abiotic stress [13]. So recombinant allergens obtained by relying on prokaryotic expression systems may not truly simulate the state of natural ones [14]. Furthermore, natural variations in post-translational cleavage timing and specificity often lead to the heterogeneous forms of allergens in plant extracts, posing a considerable challenge to the purification of homogeneous native allergen proteins [15]. Processing sites can be identified through mass spectrometry and linked to allergenicity and processing mechanisms [16].

In this study, we aimed to isolate and characterize the naturally occurring form of Pru du 8 present in mature almonds. Using one-step anion-exchange chromatography, we successfully purified a 15 kDa fragment of Pru du 8 from almond extract and confirmed its IgE-binding activity. The structural properties of this fragment were analyzed, and its B-cell epitopes were predicted using computational tools to provide insights into its allergenic potential.

## 2. Results

### 2.1. Protein Purification

**Figure 1:**
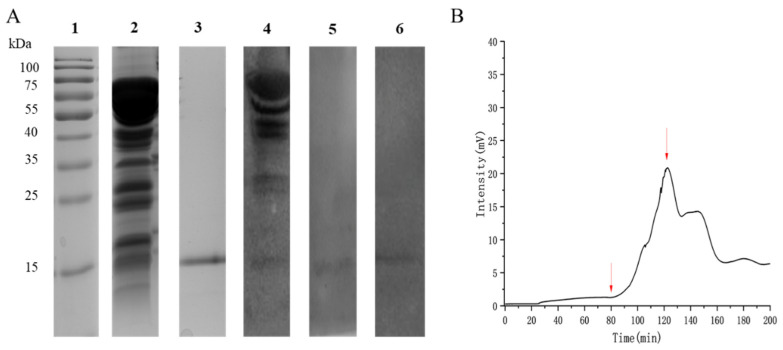
Isolation of a 15 kDa Pru du 8 Fragment from Almond Extract. (**A**) SDS-PAGE (lanes 2–3) and Western blot (lanes 4–6) analysis of almond protein extracts. Lane 1: pre-stained protein molecular weight marker. For SDS-PAGE: Lane 2, crude almond extract; Lane 3, purified 15 kDa fraction obtained by one-step anion-exchange chromatography. For Western blot: Lane 4, crude almond extract (corresponding to lane 2); Lane 5, purified 15 kDa fragment (corresponding to lane 3, probed with blood serum from allergic patient 1); Lane 6, purified 15 kDa fragment (corresponding to lane 3, probed with blood serum from allergic patient 2); (**B**) Anion-exchange chromatography profile of the crude almond protein extract on a DEAE-Sepharose Fast Flow column. The first arrow indicates sample loading; the second arrow indicates the peak fraction collected as the purified protein.

SDS-PAGE analysis of the crude extract revealed multiple 15–75 kDa protein bands (Figure 1A). Western blot analysis using blood serum from almond-allergic subjects specifically identified a protein band at approximately 15 kDa (Figure 1A), indicating its IgE-binding potential. In this study, the 15 kDa protein was isolated by purifying a crude extract through one-step anion-exchange chromatography. The sample was loaded at the point indicated by the first arrow in Figure 1B and eluted isocratically with 10 mM PBS (pH 6.5) at a flow rate of 0.5 mL/min. The target protein eluted as a single symmetrical peak, collected at the position marked by the second arrow in Figure 1B. Using the aforementioned one-step anion exchange chromatography method, approximately 3 milligrams of a 15-kDa protein fragment was obtained from 4 g of almond defatted powder. The purity of this fragment was over 95% as determined by SDS-PAGE analysis. The SDS-PAGE analysis of the purified fraction showed a single prominent band with a molecular weight of approximately 15 kDa. Western blot analysis demonstrated that the purified 15 kDa protein reacted with IgE in a blood serum from an almond-allergic individual (Figure 1A). This IgE-binding capacity was corroborated by reactivity with blood serum from a peanut-allergic individual, but no reactivity was observed with a blood serum from a non-atopic control (Supplementary Figures S1–S6).

### 2.2. Protein Identification

The protein extracts were separated by SDS-PAGE and analyzed by Western blotting. Then, 15 kDa of IgE-binding protein was excised from the gel, digested with trypsin, and identified through mass spectrometry analysis. The matching peptide yielded a MASCOT score of 289.89 for the protein A0A5E4EYT9, which was considerably higher than the scores obtained for other peptides. Nine specific peptides were detected, and the sum of the peptide molecular weights showed that the electrophoretic molecular weights were basically the same. The peptide is a potential cysteine-rich antibacterial protein with the C[X]3C−[X]10−12−C[X]3C characteristic motif of hairpin protein. These results indicate that the purified protein is Pru du 8 (Table 1).

**Figure 2:**
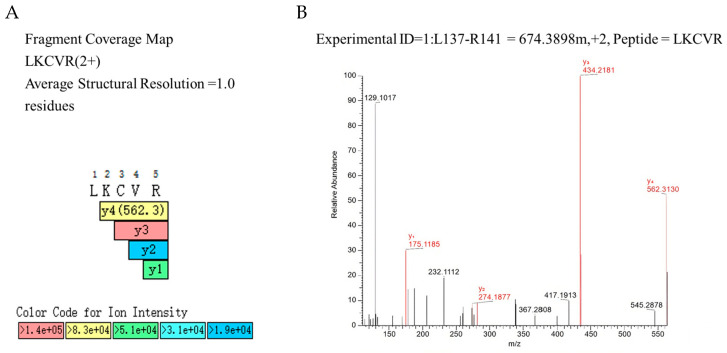
C-terminal tandem mass spectrometry analysis of the purified 15 kDa Pru du 8 fragment. (**A**) MS/MS spectrum of the C-terminal peptide. The y_1_, y_2_, y_3_, and y_4_ ions represent fragments generated by sequential cleavage from the C-terminus, confirming that the ultimate C-terminal residue is arginine (R). The color gradient indicates ion intensity, with red representing the highest intensity and blue the lowest. m/z: mass-to-charge ratio. (**B**) Schematic representation of the C-terminal peptide sequence (residues 138–142: Leu-Lys-Cys-Val-Arg) and the corresponding y-ion series. The y_1_ ion corresponds to the C-terminal arginine (R), y_2_ to Val-Arg (VR), y_3_ to Cys-Val-Arg (CVR), and y_4_ to Lys-Cys-Val-Arg (KCVR). The numbers above the sequence indicate the position of each residue in the full-length Pru du 8 protein.

However, the molecular weight of the protein we extracted was smaller than that registered in UniProt (15 kDa vs. 31 kDa Figure S7). To further characterize this protein, we performed a second round of mass spectrometry analysis, specifically C-terminal sequencing, to locate the cleavage site (Figure 2). C-terminal identification was carried out using Fourier transform mass spectrometry combined with nanoliter electrospray ionization for secondary mass spectrometry [17,18]. Peptide segments were fragmented into fragment ions through induced dissociation, and the sequence was derived by detecting the mass-to-charge ratio and intensity of the fragments [19].

The mass spectrometry data provided peptide sequencing accuracy at the single-residue level, and fragment ion information was sufficient to discriminate between adjacent amino acid residues. This feature is a hallmark of high-quality peptide identification [20]. Figure 2A shows the C-terminal sequence obtained through mass spectrometry. The color codes correspond to the intervals of ion intensity [21]. The y_1_, y_2_, y_3_, and y_4_ ions represent fragments generated by the C-terminal cleavage of the peptide during tandem mass spectrometry. These fragments are denoted by the general term yₙ, where n indicates the number of residual amino acids from the C-terminus. Specifically, the y_1_ ion corresponds to the first residue at the C-terminus [22], that is, R is the ultimate C-terminal residue. A schematic representation of the peptide sequence and the corresponding y-ion series is shown in Figure 2B.

### 2.3. Protein Characterization

**Figure 3:**
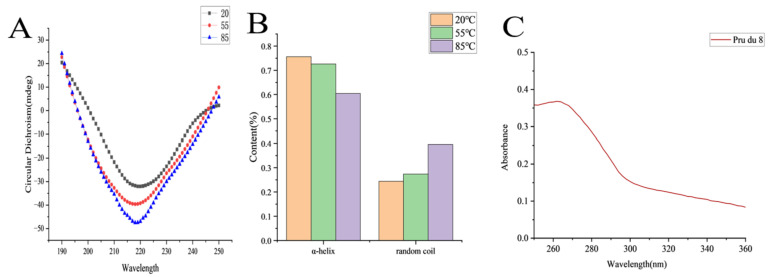
Secondary structure, thermal stability and tertiary structure analysis of the 15 kDa Pru du 8 fragment isolated from almonds. (**A**) Circular dichroism spectra of the 15 kDa Pru du 8 fragment purified at different temperatures: 20 °C (gray line), 55 °C (red line) and 85 °C (blue line). (**B**) Quantitative analysis of the secondary structure distribution of the 15 kDa protein corresponding to A. (**C**) Ultraviolet absorption spectrum of the 15 kDa Pru du 8 fragment in the range of 250 nanometers to 300 nanometers.

The secondary structure and thermal stability of the 15 kDa protein were analyzed by circular dichroism (CD) spectroscopy. The CD spectrum suggested that the protein was well-folded and exhibited a characteristic α-helical signature with a maximum at 190 nm and a minimum at 219 nm. The signature was accompanied by a distinct negative peak near 220 nm (Figure 3A). These features are consistent with typical α-helical secondary structure [23]. The CD spectrum showed no characteristic β-sheet negative absorption peak, but only featureless signals were indicative of random coils. The protein’s secondary structure comprised only α-helices (75.6%) and random coils (24.4%; Figure 3B). As temperature increased from 20 °C to 55 °C, the negative peak depth and overall spectral shape remained unchanged, indicating structural stability and minimal loss in α-helical content. Upon further heating to 85 °C, the negative peak shallowed, and the curve flattened between 210 and 230 nm, suggesting the thermal unfolding of α-helices into disordered structures and marking the onset of denaturation [24].

Ultraviolet (UV) spectroscopy revealed a pronounced absorption peak between 260 and 290 nm, indicating the presence of UV-absorbing aromatic amino acids, which are primarily tryptophan and tyrosine, in the protein (Figure 3C) [25]. The absorbance decreased gradually on both sides of the peak and plateaued beyond 300 nm, demonstrating weak absorption in the long-wavelength UV region, consistent with typical spectral characteristics of aromatic residue-containing proteins [26]. A slight blue shift was observed, suggesting that the aromatic residues were embedded within a hydrophobic core. This configuration is indicative of a compact tertiary structure. Consistent with the structural profile expected for alpha-hairpinin family proteins, the isolated 15 kDa fragment exhibited a predominantly α-helical and compact tertiary fold [27].

### 2.4. Epitope Prediction

**Figure 4:**
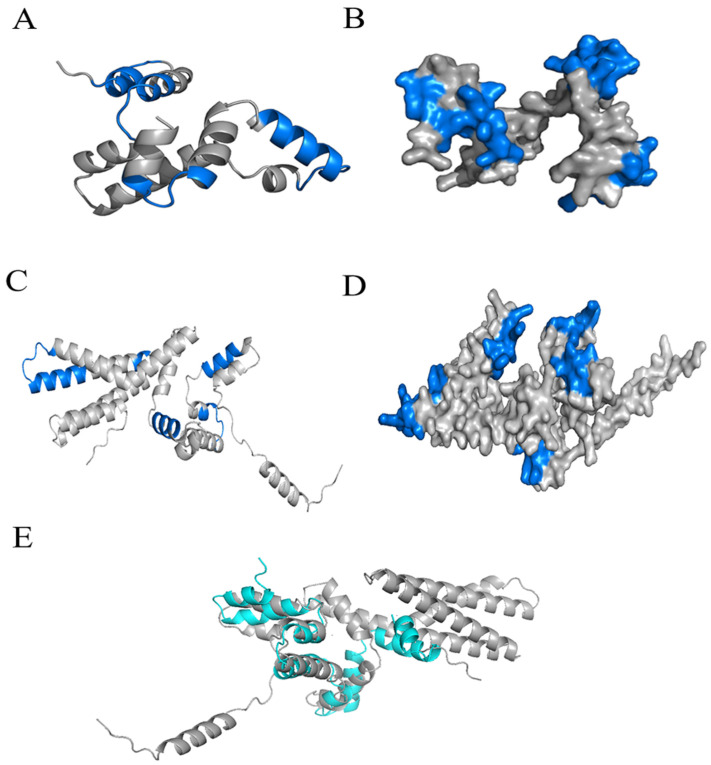
Homology modeling, structural comparison, and epitope prediction of the 15 kDa fragment and full-length 31 kDa Pru du 8 protein. (**A**) Predicted secondary structure of the 15 kDa Pru du 8 fragment. (**B**) Three-dimensional homology model of the 15 kDa fragment; blue regions indicate predicted B-cell epitopes (see Table 2 for epitope sequences). (**C**) Predicted secondary structure of the full-length 31 kDa Pru du 8 protein. (**D**) Three-dimensional homology model of the full-length protein; blue regions indicate predicted B-cell epitopes. (**E**) Superposition of the 15 kDa fragment (green) onto the corresponding region of the full-length 31 kDa protein (silver). The alignment of 669 Cα atoms yielded a root-mean-square deviation (RMSD) of 4.752 Å, indicating conserved overall fold but distinct local conformational variations between the two forms. All models were generated using the SWISS-MODEL online tool based on the amino acid sequences identified by mass spectrometry.

The allergenicity of an allergen is closely associated with its epitopes, which are specific structures recognized by IgE in the blood serum of allergic patients. Therefore, we conducted structural modeling for the extracted 15 kDa protein and the full-length 31 kDa Pru du 8 and epitope prediction analysis and compared their modeled structures [28]. The amino acid sequences identified by mass spectrometry were used to generate three-dimensional homology models of the 15 kDa fragment (Figure 4A,B) and the full-length 31 kDa protein (Figure 4C,D) using the SWISS-MODEL online tool. Predicted B-cell epitopes are shown in blue on the tertiary structures (Figure 4B,D). Superposition of the 15 kDa fragment onto the corresponding region of the full-length protein (Figure 4E) yielded an RMSD of 4.752 Å, indicating that although the overall fold is conserved, distinct local conformational variations exist between the two forms [24]. Both proteins were analyzed using five B-cell linear epitope prediction tools: IEDB, ABCpred, BcePred, ElliPro, and the Immunomedicine Group [3]. As for the 15 kDa protein, the prediction results showed high consistency across different tools, leading to the identification of four high-confidence epitopes: ^4^TCEEGCSI^12^, ^22^LQMCSSHGQ^30^, ^68^CIRSPDREMCERACQ^83^, and ^98^KMITRDPR^105^ (Table 2). These epitopes were located on the protein surface (Figure 4B). Notably, when compared with the prediction results for the full-length 31 kDa protein (Table 3), the epitope distribution pattern of the 15 kDa protein differed from that of its corresponding region in the intact protein. Although they shared the ^105^MCERACQ^111^ epitope, the surface accessibility and spatial conformation of this region were altered within the large structural context of the 31 kDa protein. Notably, the C-terminal extension of the 31 kDa protein revealed additional potential epitopes absent in the 15 kDa fragment. These structural and epitopic differences suggest that the 15 kDa fragment and its corresponding region in the full-length protein exhibit distinct immunorecognition properties. Although this fragment retains certain key epitopes, its structural state as an independent protein alters the spatial distribution and accessibility of these epitopes, providing important insights into its sensitization mechanism. All epitope information provided in this study is based on bioinformatic predictions, and their actual immunoreactivity requires further experimental validation.

## 3. Discussion

Studies of methods for extracting natural Pru du 8 from almonds are limited to date. Nevertheless, Che et al. obtained cDNA from the total RNA of almonds and determined the complete sequence of the previously reported almond allergens. The recombinant Pru du 8 sequence was determined on the basis of its cDNA sequence, but the extraction of Pru du 8 from almond or the expression product was unsuccessful [7]. In this study, we developed a one-step anion-exchange chromatography method and successfully obtained a highly pure (>95%) 15 kDa fragment of Pru du 8, confirming its IgE-binding activity. C-terminal sequencing identified the terminal residue as Arg (R), indicating that the extracted protein terminates at this position. Based on the observed molecular weight (~15 kDa vs. the theoretical 31 kDa), we hypothesize that the isolated protein represents the N-terminal fragment generated by post-translational cleavage during almond maturation. This hypothesis is further supported by a previous study by Kabasser et al., who isolated a 20 kDa IgE-binding protein identified as the C-terminal fragment of Pru du 8 from almonds [8]; together, these findings suggest that full-length Pru du 8 undergoes proteolytic processing in mature almonds. Although the current inability to isolate the intact native protein prevents us from definitively determining whether the 15 kDa fragment represents the predominant form, its reproducible isolation and consistent IgE-binding activity across multiple patient sera confirm its status as a naturally occurring and immunologically relevant fragment. Regarding its contribution to actual allergic reactions, this study provides foundational evidence of its IgE-binding capacity. Therefore, the purified 15 kDa fragment obtained in this work represents a valuable tool for future investigations—such as basophil activation tests, simulated gastric digestion assays, or larger cohort studies—which are essential for evaluating its clinical relevance and supporting the development of targeted diagnostic and therapeutic strategies for almond allergy. However, this study has several limitations. First, the blood serum samples were obtained from only two almond-allergic patients, and the selection was based on availability rather than a randomized or representative cohort. While control experiments using blood serum from a non-atopic healthy individual (Supplementary Figure S5) confirmed the specificity of the observed IgE-binding signals, the limited sample size may constrain the generalizability of the immunoreactivity findings.

The 31 kDa recombinant Pru du 8, which was cloned from unripe almond cDNA, is now recognized as a member of the alpha-hairpinin family, despite initial misclassification [7]. Its structure closely resembles homologous antifungal peptides in wheat, which consist of a signal peptide, a conserved alpha-hairpinin domain, and a C-terminal domain [12]. These peptides are known to be proteolytically cleaved from the N-terminal region of vicilin precursors. Given that this conserved processing mechanism is widespread in plants [29], Pru du 8 was initially misidentified; IgE-binding alpha-hairpinin peptides in peanut and walnut also originate from vicilin proproteins [8,30,31]. Vicilins (7S globulins), which are major seed storage proteins, are commonly cleaved during germination or storage [32,33]. We speculated that the full-length Pru du 8 proprotein may similarly undergo post-translational proteolysis in mature almonds in the same manner as antimicrobial peptides in macadamia [11].

This hypothesis supports classifying Pru du 8 as an alpha-hairpinin antimicrobial protein rather than a vicilin or 2S albumin, consistent with post-translational processing of its homologs in wheat and peanuts. However, this study is limited by its reliance on mass spectrometry and computational predictions without experimental validation of hydrolytic enzymes or epitope–IgE binding. Future research should employ competitive inhibition tests and epitope validation to confirm allergenicity and support the development of low-allergenic almond products.

## 4. Materials and Methods

### 4.1. Patient Blood Serum

Blood serum samples were collected from two patients with confirmed almond allergy after informed consent was obtained (Table 3). Blood serum samples were collected from patients with almond allergy after informed consent was obtained or purchased from Plasma Lab International (Chongqing, China, Co., Ltd., Chongqing, China; Table 3).

### 4.2. Protein Extracts

Almonds (*Prunus dulcis*, Xinjiang thin-skinned variety, sourced from Kashi, China, purchased from were ground into a fine powder under liquid nitrogen. The paste obtained after liquid nitrogen grinding was defatted with acetone (analytical grade, Sangon Biotech, China) at a ratio of 1:10 (*w*/*v*) for 3 h at room temperature. The resulting skimmed powder was stored at −20 °C until further use. Protein extracts were obtained by homogenizing the skimmed powder in 0.01 mol/L PBS buffer (pH 6.5) in a 1:10 (*w*/*v*) ratio. After magnetic stirring for 3 h at 4 °C, the supernatant was collected through centrifugation at 5000× *g* for 20 min (4 °C), filtered through a 0.45 μm membrane (Sangon Biotech, China), and stored at −20 °C. Protein concentration was determined using a Bradford protein assay kit (Sangon Biotech, China) in accordance with the manufacturer’s instructions.

### 4.3. Protein Purification

The extract (approximately 35 mg total protein) was subjected to anion-exchange chromatography on a DE-AE-Sepharose Fast Flow column (XK 16/40, column volume 65 mL, Cytiva, Marlborough, MA, USA). Elution was carried out with 10 mM PBS solution, which was prepared by dissolving 1.59 g of Na_2_HPO_4_·12H_2_O (Sangon Biotech, China) and 3.51 g of NaH_2_PO_4_·2H_2_O (Sangon Biotech, China) in 1900 mL of deionized water to a final volume of 2 L, and the pH was adjusted to 6.5. The flow rate was set at 0.5 mL/min, and fractions were collected at 3 min intervals. Equal elution was performed using 10 mM PBS (pH 6.5) as the buffer. This was done because the preliminary experiments indicated that the target protein could be effectively eluted under these conditions, and a salt gradient was not necessary. All components collected during the elution process (1.5 mL per tube) were analyzed using SDS-PAGE. The corresponding components were combined for subsequent analysis.

### 4.4. SDS-PAGE Analysis

Protein samples obtained from different stages of the purification procedure were analyzed by SDS-PAGE. Specifically, the crude almond extract (prepared as described in Section 4.2) and the purified 15 kDa fragment (collected from the peak fraction in Section 4.3) were mixed with 5× SDS loading buffer (containing 250 mM Tris-HCl pH 6.8, 10% SDS, 30% glycerol, 5% β-mercaptoethanol, 0.02% bromophenol blue; all from Sigma-Aldrich, St. Louis, MO, USA) at a 5:1 ratio and heated at 100 °C for 5 min. Electrophoresis was carried out on 4–15% gradient SDS-polyacrylamide gels (Mini-PROTEAN TGX Precast Gels, Bio-Rad, Hercules, CA, USA) using a Mini-PROTEAN Tetra System (Bio-Rad, Hercules, CA, USA) with Tris-SDS running buffer. Pre-stained molecular weight markers (PageRuler Plus, Thermo Scientific, Waltham, MA, USA) were used as references. After electrophoresis, gels were stained with Coomassie Blue solution (composition detailed in Section 4.2) and destained overnight. Gel images were captured using a ChemiDoc XRS+ imaging system (Bio-Rad, Hercules, CA, USA) and analyzed with Quantity One software (v4.6, Bio-Rad).

### 4.5. Western Blot Analysis of IgE-Binding Capacity of Almond Protein Samples

The IgE-binding capacity of the crude almond extract (prepared as described in Section 4.2) and the purified 15 kDa fragment (collected from the peak fraction in Section 4.3) was assessed by Western blot. Protein samples were first separated by SDS-PAGE under reducing conditions (as described in Section 4.4) and then transferred to a PVDF membrane for immunodetection. After SDS-PAGE separation, proteins were transferred by electrophoretic transfer to a polyvinylidene fluoride membrane (Bio-Rad, Shanghai, China). The membrane was blocked with 5% bovine blood serum albumin for 2 h at room temperature and subsequently incubated overnight at 4 °C with serum samples (diluted 1:100 in TBST) from almond-allergic patients, a peanut-allergic patient, or a healthy individual. The almond-allergic patient sera were collected from patients diagnosed at the Second Affiliated Hospital of Nanchang University (as described in Section 4.1, Table 3), while the peanut-allergic and healthy control sera were purchased from PlasmaLab International (Chongqing, China). Detection was carried out using horseradish peroxidase–conjugated goat anti-human IgE antibody (diluted 1:5000 Bio-Rad, Hercules, USA), followed by enhancement with streptavidin (Neobioscience, Beijing, China). Between each step, the membrane was washed five times for 5 min each with TBST. Immunoreactive bands were visualized with enhanced chemiluminescence reagent (Sangon Biotech, Shanghai, China), and chemiluminescent signals were captured and quantified after 1 min of reaction with an ImageQuant LAS 500 imaging system (IMH-bio Technology, Beijing, China).

### 4.6. Mass Spectrometry

#### 4.6.1. Protein Identification Through Mass Spectrometry

The 15 kDa protein bands were excised from the SDS-PAGE gel for mass spectrometric identification. After reduction, alkylation, in-gel digestion, and desalting according to established protocols [16], peptides were analyzed using an EASY-nLC 1200 system coupled to a Q Exactive mass spectrometer (Thermo Scientific, USA) with a one-dimensional analytical column. The acquired spectra were searched against the UniProt database with Proteome Discoverer software (version 2.5, Thermo Scientific, USA).

#### 4.6.2. C-Terminus Identification Through Mass Spectrometry

For C-terminal sequencing, the 15 kDa protein band corresponding to the purified Pru du 8 fragment (from the SDS-PAGE gel shown in Figure 1A, lane 3) was excised and destained with 50 mM NH_4_HCO_3_ in 50% acetonitrile until the Coomassie Blue coloration was completely removed. After reduction with 10 mM DTT at 56 °C for 1 h and alkylation with 20 mM iodoacetamide in the dark at room temperature for 1 h, the gel pieces were digested separately with trypsin (0.025 μg/μL) and chymotrypsin (0.05 μg/μL) at 37 °C and 30 °C, respectively, for 16 h. After enzymatic digestion, the peptides were extracted, desalted using C18 ZipTips, redissolved in 0.1% formic acid/2% acetonitrile, and subjected to mass spectrometric analysis. Data acquisition was performed using a Vanquish Neo system coupled to an Orbitrap Fusion Lumos high-resolution mass spectrometer (Thermo Scientific), and the data were processed with Thermo BioPharma Finder software (version 5.2, Thermo Scientific, Waltham, MA, USA).

### 4.7. Circular Dichroism (CD) Spectroscopy

CD spectra were recorded using an MOS-450 spectropolarimeter (Bio-Logic SAS, Claix, France) with a 1 mm path-length quartz cuvette. The purified 15 kDa Pru du 8 fragment was prepared at 0.2 mg/mL in 10 mM PBS (pH 6.5). Spectra were collected from 190 to 250 nm at a scan speed of 50 nm/min, with each spectrum representing the average of three scans at 20 °C.

### 4.8. Thermal Stability

To assess the thermal stability of the purified 15 kDa Pru du 8 fragment, CD spectra were recorded at 20 °C, 55 °C, and 85 °C under the same conditions described in Section 4.7. Changes in secondary structure content were monitored by analyzing the spectral variations at different temperatures.

### 4.9. Ultraviolet (UV) Spectrometer

UV absorption profiles were recorded from 250 nm to 300 nm with a UV spectrophotometer (Purkinje General, Beijing, China) for samples (allergen solutions were prepared at a concentration of 0.2 mg/mL in PBS) dissolved under the same buffer conditions.

### 4.10. Structure Simulation and Epitope Prediction

To compare the structural features and epitope distribution of the purified fragment with the intact allergen (as presented in Section 2.4 and Figure 4), the amino acid sequence of the purified 15 kDa Pru du 8 fragment, as determined by mass spectrometry (see Section 2.2 and Table 1), and the full-length sequence of Pru du 8 (UniProt ID: A0A5E4EYT9) were submitted separately to the SWISS-MODEL online tool for homology modeling. “Then, the model with the highest GMQE score (closest to 1) and the best QMEANDisCo value (closest to 0) was selected for secondary and tertiary structure prediction. Linear and conformational B-cell epitopes were predicted using five online tools: IEDB (http://tools.iedb.org/ (accessed on 12 May 2025)), ABCpred (https://webs.iiitd.edu.in/raghava/abcpred/ (accessed on 12 May 2025)), ElliPro (http://tools.iedb.org/ellipro/ (accessed on 12 May 2025)), BcePred (https://webs.iiitd.edu.in/raghava/bcepred/ (accessed on 12 May 2025)), and Immunomedicine Group (http://imed.med.ucm.es/Tools/ (accessed on 12 May 2025)). Epitopes that were consistently predicted by at least three of the five tools were considered the most reliable and selected for further analysis [23]. The selected epitopes were visualized on the simulated tertiary structure using PyMOL (Version 3.1.0, Schrödinger, Inc., New York, NY, USA), and their spatial distribution was analyzed.

## Figures and Tables

**Table 1:** The 15 kDa protein band was excised from the SDS-PAGE gel, digested with trypsin, and analyzed by LC-MS/MS. Nine unique peptides matching the cysteine-rich antibacterial protein A0A5E4EYT9 (Pru du 8) were identified. The high MASCOT score (289.89) and multiple matched peptides confirm the identity of the purified protein as Pru du 8. Start and termination sites are numbered according to the amino acid sequence of the full-length Pru du 8 protein (UniProt A0A5E4EYT9).

Start Site	Termination Site	Peptide Sequences	Theo. MH+ [Da]
30	46	AQVTCEEGCYSISDQSK	1961.82
47	64	VGECLQMCSSHGQSCEDR	2155.82
71	80	WPQQQEQCLR	1372.64
84	94	QQEQGHHLPCR	1389.64
84	99	QQEQGHHLPCREQCIR	2075.96
100	108	SPDREMCER	1179.48
109	120	ACQQQQGQGGGR	1274.56
109	125	ACQQQQGQGGGRQCLQR	1959.89
135	141	ERLKCVR	960.54

**Table 2:** Comparison of linear B-cell epitopes predicted using five independent bioinformatics tools (IEDB, ABCpred, BcePred, ElliPro, and Immunomedicine Group) for the 15 kDa fragment and the full-length 31 kDa Pru du 8 protein, showing the amino acid position ranges identified by each method and the consensus epitopes supported by multiple prediction approaches.

	ABCpred		Immunomedicine Group		Bcepred		Elipro		IEDB		Predict Epitopes	
	15 kDa	31 kDa	15 kDa	31 kDa	15 kDa	31 kDa	15 kDa	31 kDa	15 kDa	31 kDa	15 kDa	31 kDa
1	^5^C-E^21^		^4^T-I^12^		^9^Y-V^17^		^1^A-T^4^		^6^E-G^19^		^4^T-I^12^	
2	^22^L-M^38^		^17^K-Q^30^		^22^Q-E^32^	^44^Q-S^55^	^7^E-K^17^		^26^S-C^49^		^22^L-Q^30^	
3	^28^H-P^44^	^47^V-C^61^	^45^Q-M^52^	^38^C-K^46^	^67^R-E^73^	^55^S-R^64^	^40^A-Q^48^	^49^E-S^60^	^45^R-Q^92^	^49^E-W^71^	^68^C-Q^83^	^47^V-S^55^
4	^67^Q-Q^83^	^65^C-E^76^	^58^Q-R^70^	^58^G-P^72^	^81^C-R^90^	^109^A-C^122^	^68^C-A^80^	^67^R-C^78^	^98^K-L^108^	^65^C-Q^87^	^98^K-R^105^	^65^C-P^72^
5		^105^M-G^118^		^88^G-C^97^		^134^R-F^152^		^99^R-Q^113^		^88^G-C^122^		^105^M-Q^113^
6		^145^Q-V^161^		^115^G-I^129^		^167^R-Q^192^		^127^K-Q^145^		^169^C-R^199^		^169^C-Q^188^
7		^190^M-R^208^		^156^Q-E^172^		^204^W-Q^212^		^167^R-Q^188^		^201^R-C^219^		^201^R-R^208^

**Table 3:** Information on patients allergic to almonds.

Patient No.	Gender	Age (Years)	Clinical Symptoms	Total IgE Level (KU/L)	Specific IgE Level (KU/L)
1	Female	21	Allergic rhinitis, no immunotherapy	2516	41.2
2	Male	30	Allergic rhinitis, currently undergoing immunotherapy	1109	27.4

## Data Availability

All data generated and analyzed during this study are included in this published article and its Supplementary Materials.

## Supplementary Materials

The following supporting information can be downloaded at: https://www.mdpi.com/article/10.3390/ijms27062683/s1.

## Abbreviations

The following abbreviations are used in this manuscript:

WB	Western Blot
PBS	Phosphate-buffered saline
CD	Circular dichroism
PVDF	Polyvinylidene fluoride
UV	ultraviolet
M	mol/L
IgE	immunoglobulin E
kDa	kilodalton
(WHO/IUIS)	World Health Organization/International Union of Immunological Societies
Theo. MH^+^ [Da]	theoretical monoisotopic mass of the protonated peptide
