# Supplementary material for: Identification, Characterization and Epitopes Prediction of an Almond Allergen Pru du 8 Fragment
Source: Int J Mol Sci. 2026 Mar 15;27(6):2683. doi: 10.3390/ijms27062683 (PMC13027201; doi:10.3390/ijms27062683)
Supplement: Supplementary file 1 [file ijms-27-02683-s001.zip › ijms-4132851-supplementary.pdf]

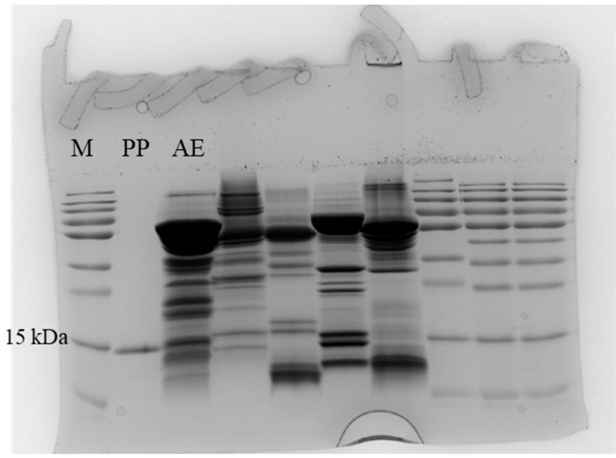

Figure S1. Unprocessed SDS-PAGE gel images of almond protein extraction and purification.
Lane M: pre-stained protein molecular weight marker; lane AE: crude almond extract (total proteins extracted from defatted almond powder); lane PP: purified 15 kDa protein fraction obtained after one-step anion-exchange chromatography. This image demonstrates the purity of the isolated fragment and serves as the original data for Figure 1A. Unlabeled lanes correspond to samples unrelated to the present study and are shown here only to demonstrate the integrity of the original gel data.

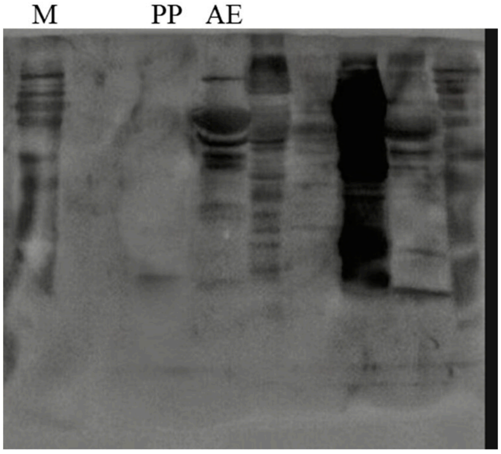

Figure S2. Unprocessed Western blot images using serum from almond-allergic Patient 1.
After SDS-PAGE and electrophoretic transfer to PVDF membrane, the blot was probed with serum (diluted 1:100) from an almond-allergic patient (Patient 1, female, age 21). Detection was performed using HRP-conjugated anti-human IgE antibody. Lane M: protein marker; lane AE: almond extract; lane PP: purified 15 kDa protein. The arrow indicates the specific IgE-reactive band at approximately 15 kDa, confirming the allergenicity of the purified fragment. Unlabeled lanes correspond to samples unrelated to the present study and are shown here only to demonstrate the integrity of the original Western blot data.

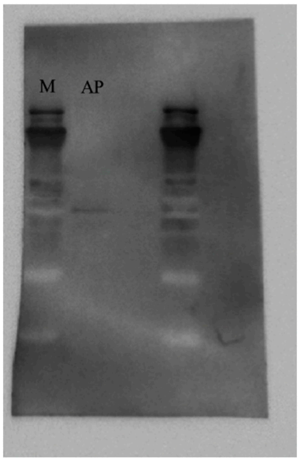

Figure S3. Unprocessed Western blot images using serum from almond-allergic Patient 2.
The blot was probed with serum (diluted 1:100) from a second almond-allergic patient (Patient 2, male, age 30) to further validate IgE-binding capacity. Lane M: protein marker; lane AE: almond extract; lane PP: purified 15 kDa protein. A consistent immunoreactive band at 15 kDa confirms the reproducibility of the IgE-binding activity across different allergic individuals. Unlabeled lanes correspond to samples unrelated to the present study and are shown here only to demonstrate the integrity of the original Western blot data.

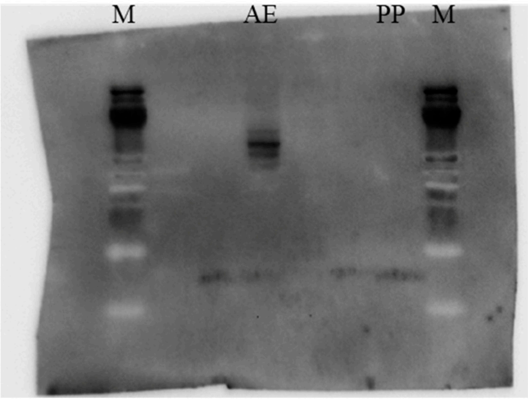

Figure S4. Unprocessed Western blot images using serum from a peanut-allergic patient.
To further assess the potential allergenicity of the extracted proteins, the peanut allergic patient serum (diluted 1:100) was used for incubation. Lane M: Protein marker; Lane AE: Raw almond extract; Lane PP: Purified 15 kDa protein. A reaction band was observed at 15 kDa, indicating that the extracted proteins have potential allergenicity. Unlabeled lanes correspond to samples unrelated to the present study and are shown here only to demonstrate the integrity of the original Western blot data.

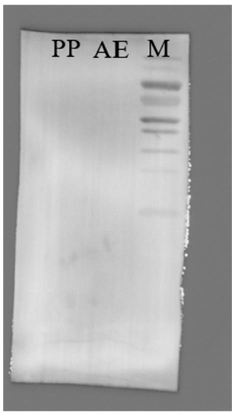

Figure S5. Unprocessed Western blot images using serum from a non-atopic healthy individual.
Negative control experiment using serum (diluted 1:100) from a healthy individual with no history of allergies. Lane M: protein marker; lane AE: almond extract; lane PP: purified 15 kDa protein. No immunoreactive bands were detected, confirming the specificity of the IgE-binding observed with allergic patient Unlabeled lanes correspond to samples unrelated to the present study and are shown here only to demonstrate the integrity of the original Western blot data. sera.

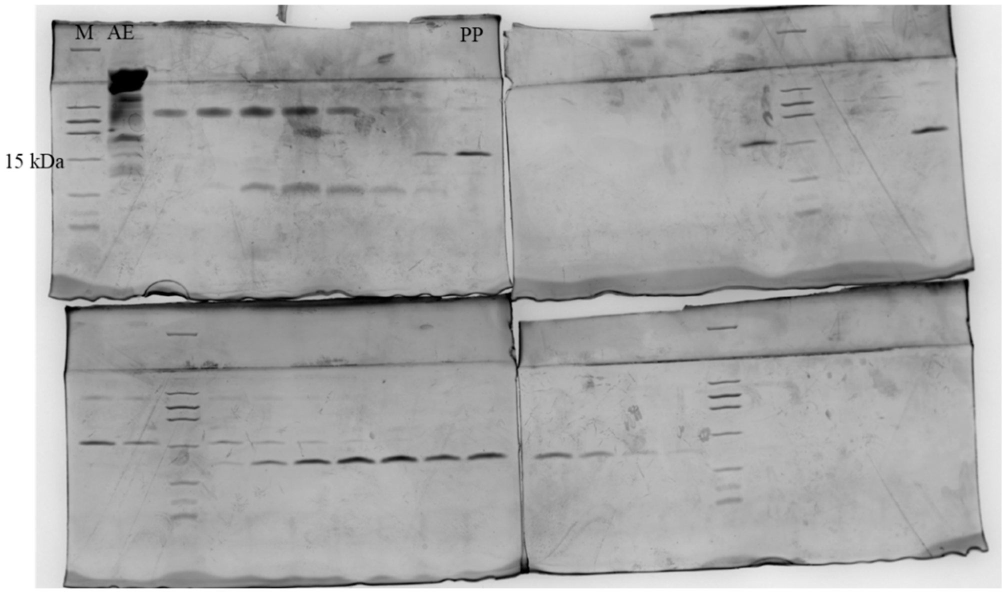

Figure S6. SDS-PAGE analysis of fractions collected during anion-exchange chromatography.
Fractions corresponding to the elution peak in Figure 1B were collected and analyzed by SDS-PAGE to monitor the purification process. Lane M: protein marker; lane AE: crude almond extract (starting material); lanes 1-8: sequential fractions collected across the chromatographic peak; lane PP: final pooled purified 15 kDa protein. The gel demonstrates the enrichment and isolation of the target protein through a single chromatographic step. Unlabeled lanes correspond to samples unrelated to the present study and are shown here only to demonstrate the integrity of the original gel data.

```
31kDa      MATMTKAELPLLVLFLSTLLLATSVPSVRAQVTCEEGCYSISDQSKVGECLQMCSSHGQS 60
15kDa      -----------------------------AQVTCEEGCYSISDQSKVGECLQMCSSHGQS 31

31kDa      CEDRCMREARWPQQQEQCLRMCRQQEQGHHLPCREQCIRSPDREMCERACQQQQGQGGGR 120
15kDa      CEDRCMREARWPQQQEQCLRMCRQQEQGHHLPCREQCIRSPDREMCERACQQQQGQGGGR 91

31kDa      QCLQRCKMITRDPRERLKCVRTCTQGQQQGVFQGQQQQQVEQQCRQHCQSERDPMRQQE 180
15kDa      QCLQRCKMITRDPRERLKCVR-------------------------------------- 112

31kDa      PQQQECQRMCREQFEQGQGIRMVA     264
15kDa      ------------------------     112
```

Figure S7. Amino acid sequence alignment of the purified 15 kDa fragment and the full-length 31 kDa Pru du 8 protein.

The sequence of the 15 kDa fragment identified by mass spectrometry was aligned with the full-length Pru du 8 sequence (UniProt A0A5E4EYT9) using ClustalW. The alignment shows that the 15 kDa fragment corresponds to the N-terminal region (residues 30-141) of the full-length protein, with complete sequence coverage in the overlapping region. The cleavage site after residue 141 (Arg) was confirmed by C-terminal sequencing (Figure 2).

C-terminal identification mass spectrometry experiments and related specific parameters

The target protein bands were excised and cut into 1 mm^3 gel pieces, which were transferred to 1.5 mL EP tubes. The gel pieces were destained with 1 mL of 50% ACN/50 mM NH_4HCO_3 until complete discoloration, followed by dehydration with 1 mL of 100% ACN until the gels shrank and turned white. After discarding the ACN, the samples were air-dried at room temperature. For reduction and alkylation, the gel pieces were treated with 100 μL of 10 mM DTT at 56°C for 1 h, followed by 100 μL of 20 mM IAM in the dark at room temperature for 1 h. The gel pieces were then washed with 500 μL of destaining solution and dehydrated again with 1 mL of 100% ACN before final drying. For enzymatic digestion, the dried gel pieces were reswelled with 100 μL of trypsin (0.025 μg/μL) and incubated at 37°C for 16 h, or alternatively with 100 μL of chymotrypsin (0.05 μg/μL) at 30°C for 16 h. Peptides were extracted twice with 200 μL of extraction buffer (5% TFA/50% ACN/45% H_2O) at 37°C for 1 h each, with sonication and centrifugation. The combined extracts were vacuum-dried at 57°C, desalted using self-packed C18 desalting columns, and concentrated in a vacuum centrifuge at 45°C. The final peptides were reconstituted in sample solvent (0.1% formic acid, 2% acetonitrile), vortexed thoroughly, centrifuged at 13,200 rpm for 10 min at 4°C, and the supernatant was transferred to vials for LC-MS/MS analysis.

For LC-MS/MS analysis, a Vanquish Neo system coupled to an Orbitrap Fusion Lumos

mass spectrometer was employed. Chromatographic separation used a 150 μm × 170 mm column packed with Reprosil-Pur 120 C18-AQ (1.9 μm) with mobile phase A (0.1% formic acid in water) and B (0.1% formic acid in 80% acetonitrile). The gradient was: 0-4 min, 4-8% B; 4-35 min, 8-28% B; 35-55 min, 28-40% B; 55-56 min, 40-95% B; 56-66 min, 95% B at 0.6 μL/min. Full MS parameters included: resolution 120,000, AGC target 3e6, maximum IT 20 ms, scan range 300-1800 m/z. dd-MS2 settings were: resolution 15,000, standard AGC target, and stepped NCE 30. Data processing was performed using Thermo BioPharma Finder with fixed modification of carbamidomethyl (C), variable modifications of oxidation (M) and acetyl (protein N-term), and enzymes specified as trypsin and chymotrypsin against the target protein sequence database.

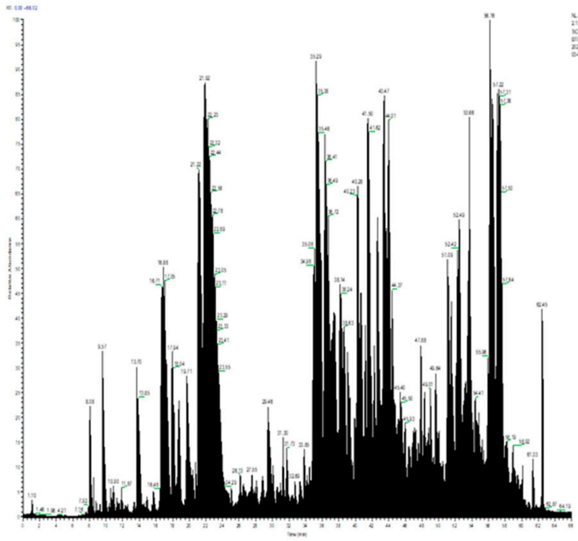

Trypsin enzyme digestion TIC map

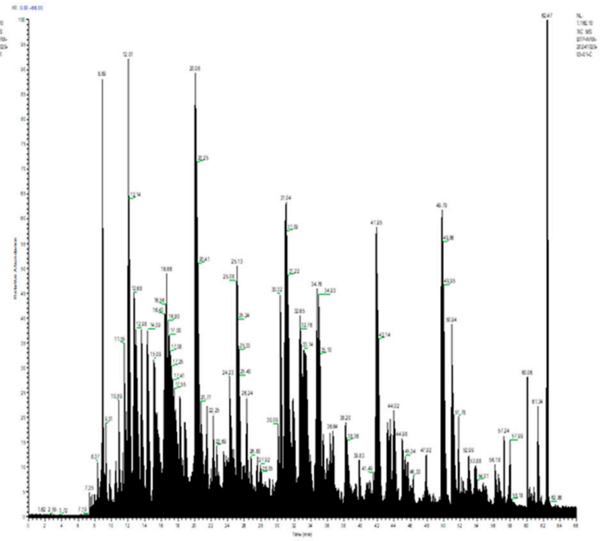

Chymotrypsin enzymatic cleavage TIC map
